# P-1862. Telehealth in Outpatient Parenteral Antimicrobial Therapy (OPAT): Utilization Patterns and Patient Outcomes

**DOI:** 10.1093/ofid/ofaf695.2031

**Published:** 2026-01-11

**Authors:** Kylie Lewis, Angela Perhac, Michael Swartwood, Teresa M Oosterwyk, Claire E Farel, Mary Catherine Bowman, Asher J Schranz

**Affiliations:** UNC Health, Chapel Hill, NC; University of North Carolina Medical Center, Chapel Hill, North Carolina; University of North Carolina Medical Center, Chapel Hill, North Carolina; UNC Medical Center, Chapel Hill, North Carolina; UNC Chapel Hill, Chapel Hill, North Carolina; UNC, Chapel Hill, North Carolina; University of North Carolina, Chapel Hill, NC

## Abstract

**Background:**

Since the COVID-19 pandemic, use of telehealth has grown. Data are limited related to use of telehealth in infectious diseases (ID), particularly for outpatient parenteral antimicrobial therapy (OPAT). We assessed the frequency of telehealth in OPAT and its association with adverse outcomes.

**Figure 1.**
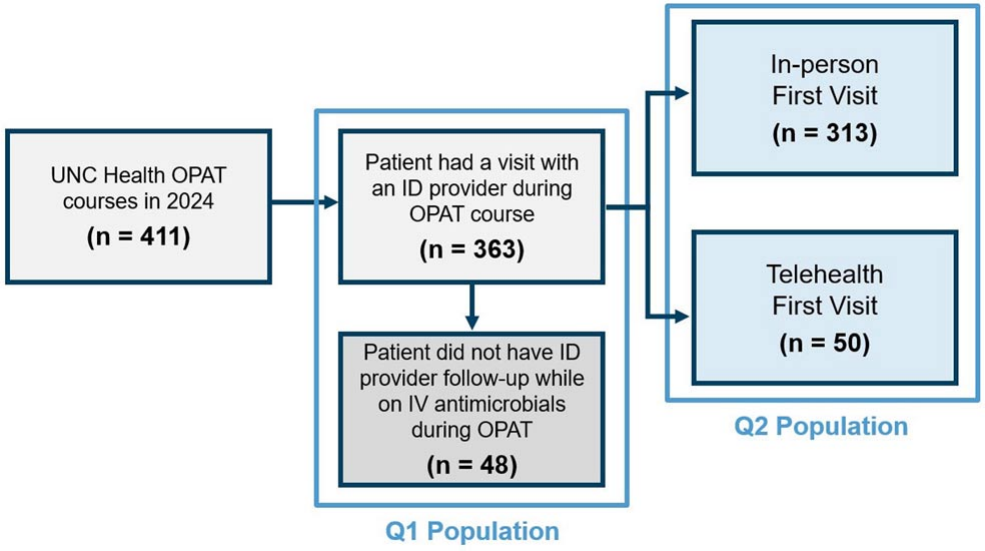
Flow diagram for OPAT course inclusion

**Table 1.**
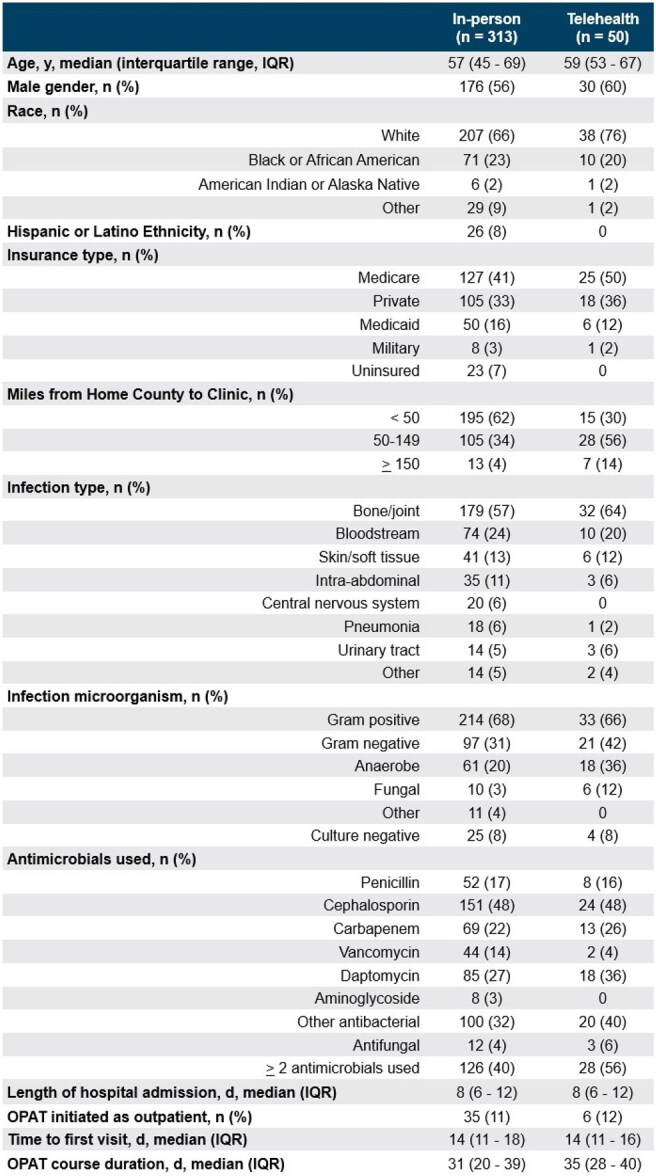
Patient characteristics for Q2 population

**Methods:**

We studied a large academic OPAT program to ask: how often is telehealth used for ID visits during OPAT (Q1), and what is the association of telehealth with an outcome of either emergency department (ED) visit or OPAT-/infection-related hospital admission (Q2)? Follow-up began at the time of the first ID visit during OPAT, at which point patients were classified as exposed to either “telehealth” or “in-person” visits, and ended at time of first outcome or OPAT completion (i.e., discontinuation of IV antimicrobials), whichever came first. We included all OPAT courses in 2024 during which an ID visit occurred. The proportion of first visits and overall visits via telehealth were tabulated. The association between telehealth vs. in-person visits and the outcome was assessed by survival analyses, using an Aalen-Johansen estimator to account for OPAT completion as a competing event.

**Figure 2.**
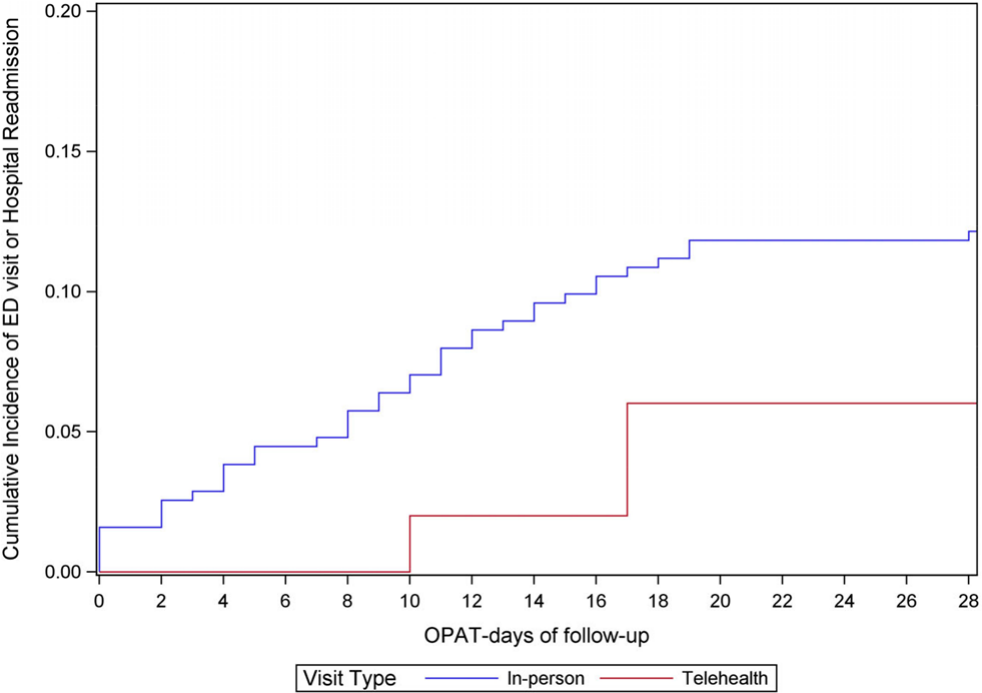
Association between first visit type and the composite outcome Survival analysis graphs comparing in-person vs. telehealth first visits with composite ED visit or OPAT/infection-related hospital readmission occurring after visit. Follow-up begins after the date of the first visit. This data shows an increased risk of the outcome for the in-person group compared to telehealth at 7 days of follow up (RD 5% [95% CI: 3%, 8%]), 14 days of follow up (RD 8% [95% CI: 0%, 11%]), and 28 days of follow up (RD 6% [95% CI: -3%, 13%]).

**Results:**

Of 411 OPAT courses, 313 (76%) had a first ID visit in-person, 50 (12%) via telehealth, and 48 (12%) did not have a visit (Figure 1). In total, 625 visits occurred during OPAT, of which 79 (13%) were telehealth. Compared to the in-person group, the telehealth group were more often white (76% vs 66%), insured (100% vs 93%), resided ≥ 50 miles from the ID clinic (70% vs 38%) and less often treated with vancomycin (4% vs 14%) (Table 1). Of 363 courses who had an ID visit during OPAT, there were 46 outcomes. The crude risk difference for the outcome at 7, 14 and 28 days after the first visit was 5% (95% CI: 3%, 8%), 8% (95% CI: 0%, 11%), and 6% (95% CI: -3%, 13%) greater for the in-person group, compared with telehealth (Figure 2).

**Conclusion:**

At a large OPAT program, telehealth was used in a minority of visits and was not associated with more ED visits or readmissions, with fewer events occurring soon after the first visit. Patients receiving telehealth have a unique demographic and clinical profile, suggesting potential confounding. Further research is needed to evaluate these findings and assess when telehealth is best utilized in OPAT.

**Disclosures:**

Asher J. Schranz, MD, MPH, Uptodate: payment for authorship

